# Mendelian randomization analysis reveals causal relationship between allergic diseases and influenza

**DOI:** 10.1016/j.waojou.2025.101077

**Published:** 2025-06-07

**Authors:** Hui Li, Yanping Zong, Lei He, Yujie Sun, Weibing Shi, Jinchen Guo

**Affiliations:** a Center for Xin'an Medicine and Modernization of Traditional Chinese Medicine of IHM, Anhui University of Traditional Chinese Medicine, Hefei 230012, China; b The First Affiliated Hospital of Anhui University of Chinese Medicine, Hefei 230031, China

**Keywords:** Allergic disease, Atopic dermatitis, Allergic rhinitis, Asthma, Influenza, Mendelian randomization

## Abstract

**Background:**

Allergic diseases and influenza share similar genetic backgrounds and pathophysiological mechanisms. Observational studies have established a correlation between these 2 conditions; however, the precise direction of the causal relationship remains unclear. This Mendelian randomization (MR) study aims to evaluate the causal relationship between allergic diseases and influenza.

**Materials and methods:**

This study utilized summary statistical data from genome-wide association studies (GWAS) and employed the two-sample MR method to comprehensively analyze the causal relationships between allergic diseases (asthma, hay fever, eczema), atopic dermatitis (AD), hay fever or allergic rhinitis (AR), and different types of influenza (including all influenza, regular influenza excluding pneumonia, and severe influenza that encompasses both influenza and pneumonia) using genetic factors as instrumental variables. The analysis primarily relied on the inverse variance weighted random effects model (IVW-RE).

**Results:**

The IVW-RE analysis revealed significant correlations between allergic diseases (asthma, hay fever, or eczema) and both all influenza and severe influenza (influenza and pneumonia). Additionally, AR (hay fever or allergic rhinitis) was associated with both all influenza and regular influenza (excluding pneumonia). Furthermore, a significant correlation was found between asthma and severe influenza (influenza and pneumonia). However, there is no evidence to support a causal relationship between AD and influenza.

**Conclusion:**

The results of this MR study support a causal relationship between allergic diseases, asthma, and influenza, including severe influenza. This finding suggests that allergic diseases and asthma are significant risk factors for influenza. Additionally, this study provides high-quality causal evidence that can inform clinical practices aimed at preventing the onset of influenza, particularly in populations with respiratory allergies and asthma.

## Introduction

Allergic diseases are common chronic immune disorders that typically manifest during childhood and exhibit significant genetic predisposition.^1^ These include atopic dermatitis (AD), allergic asthma (AAS), and allergic rhinitis (AR), among others. Hypofunctional allergic diseases affect a substantial portion of the global population, with their incidence sharply increasing over the past 3 decades, thereby imposing a considerable burden on society.^2^ The World Allergy Organization (WAO) has classified them as “public health problems of global concern”.^3^ AD, commonly referred to as eczema, is a chronic inflammatory skin condition characterized by pruritus, scaling, and crusting lesions primarily located on flexor muscles.^4^ Asthma represents a prevalent chronic airway inflammatory disorder, with AAS being its most common phenotype.^5^ Upon triggering, asthma can lead to recurrent wheezing, chest tightness, shortness of breath, and increased mucus production, potentially resulting in severe health complications. Key characteristics typically include elevated serum immunoglobulin E (IgE) levels, eosinophilic inflammation, mucus cell hyperplasia, airway hyperresponsiveness (AHR), and airway wall remodeling, which encompasses smooth muscle cell proliferation, subepithelial fibrosis, and angiogenesis.^6^ AR, also known as hay fever, is one of the most prevalent upper respiratory conditions and often coexists with chronic lower respiratory diseases (CLRDs), particularly asthma. It is characterized by non-infectious chronic inflammation of the nasal mucosa due to allergen exposure, with typical symptoms including paroxysmal sneezing, tearing, rhinorrhea, nasal itching, and nasal congestion.^7^^,^^8^

Influenza poses a significant global health challenge, often characterized by a high incidence rate and mortality, particularly among high-risk populations. The influenza virus, a member of the Orthomyxoviridae family, is a multi-segmented, single-stranded negative-sense RNA virus.^9^ Owing to the limited proofreading capabilities of its RNA polymerase, the virus exhibits a high mutation rate. Influenza viruses are categorized into types A, B, C, and D based on their genetic makeup.^10^ Among these, influenza A is known for causing the most severe illnesses in humans due to its antigenic variability. Influenza A strains are further distinguished by the surface antigens hemagglutinin (HA) and neuraminidase (NA) present on their surface.^11^ The high infectivity and rapid transmission rate of influenza viruses contribute to annual global pandemics.

The triad of asthma, AR, and AD shares common genetic and pathophysiological characteristics. Up to 80% of children with AD are likely to develop asthma or AR later in life.^12^ Furthermore, the incidence of AD is higher in adolescents with asthma compared to those without.^13^^,^^14^ Respiratory viruses can infect individuals of all ages, with pediatric populations being the most vulnerable. These respiratory viruses interact with allergens and other microorganisms through various mechanisms, promoting the development of recurrent virus-induced wheezing and asthma. Evidence has confirmed a correlation between respiratory syncytial virus (RSV) and rhinovirus (RV) infections and the onset of asthma.^15^^,^^16^ The interaction between influenza A virus (IAV) and asthma remains unclear. A prospective study conducted during the 2009 influenza pandemic indicated that H1N1 preferentially infected asthma patients compared to those without asthma. Asthma was identified as the most prevalent underlying condition associated with hospitalization due to pH1N1.^17^ Furthermore, clinical observations have revealed that patients with AR and asthma experience more severe symptoms during viral upper respiratory tract infections (URI) than individuals without allergic symptoms who are infected by the same virus under similar circumstances.^18^ In a population-based study, AD was linked to an increased risk of systemic infections, including pneumonia and influenza. For a long time, it has been believed that viral infections exacerbate asthma across all age groups. However, a global analysis of pH1N1 cases reveals that hospitalized asthma patients exhibit a higher survival rate than those with other underlying conditions, and the incidence of influenza among hospitalized asthma patients is lower compared to non-asthmatic patients.^19^ The above contradictory clinical research results further complicate the impact of allergic diseases and asthma on the incidence rate and prognosis of influenza, and this relationship needs further research.

Inferring causality between allergic diseases and influenza from traditional epidemiological studies is challenging due to the potential for confounding variables and reverse causation. Limitations related to medical ethics, experimental design, and research costs further hinder the feasibility of conducting randomized controlled trials to explore the causal effects of these conditions. In this context, MR studies can address these research limitations and provide reliable evidence for causal relationships. MR employs 1 or more genetic variations, usually single nucleotide polymorphisms (SNPs), as instrumental variables (IVs) to assess causal relationships.^20^ These genetic variations are determined at birth and remain unaffected by confounding factors, effectively reducing the influence of confounding and reverse causation on the analysis.

## Methods

### Study design

**Fig. 1:**
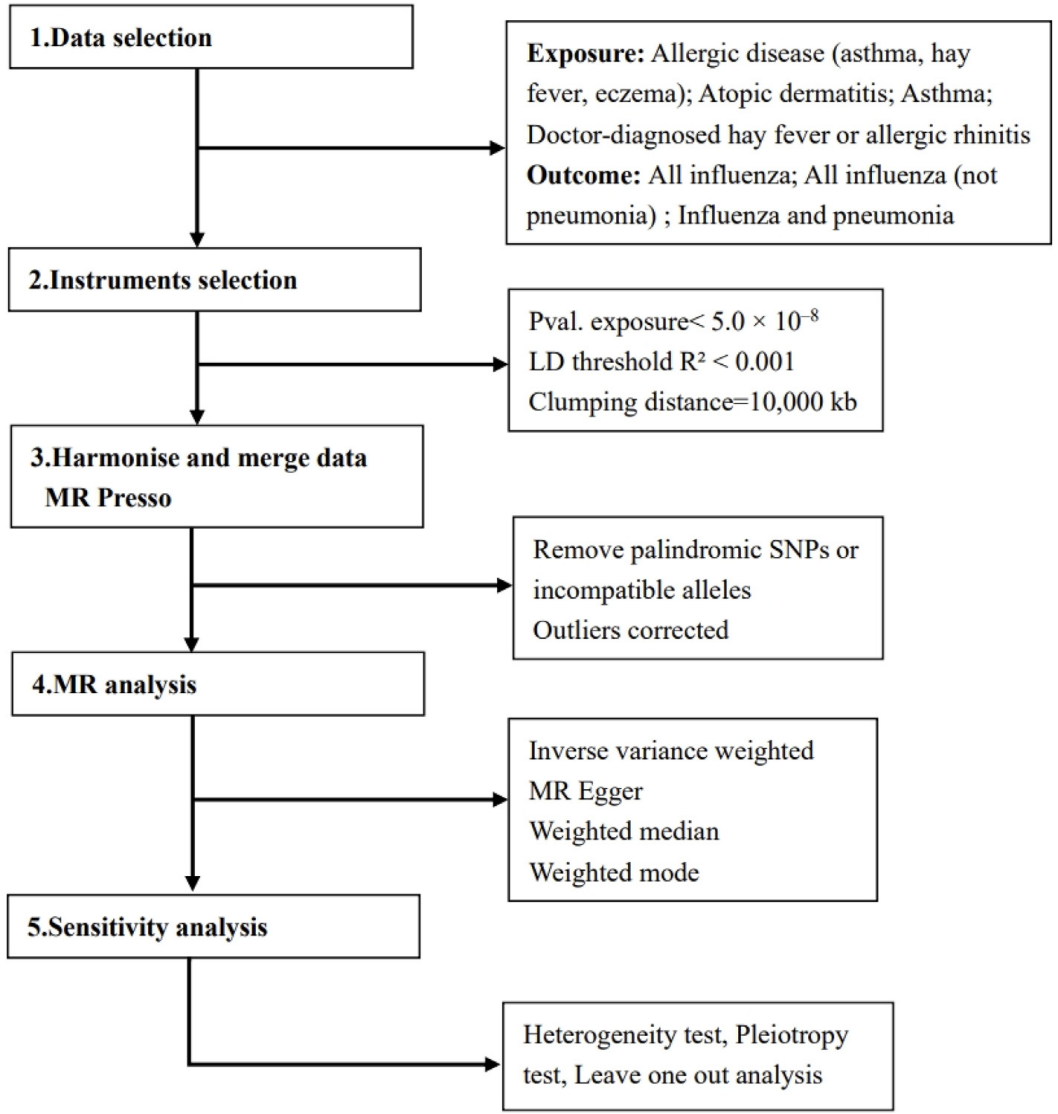
Flowchart of the MR study design. Abbreviations: MR, Mendelian randomization; SNP, single nucleotide polymorphism.

We performed a two-sample MR analysis to investigate the causal association between allergic diseases, asthma, and influenza using a compiled statistical dataset from genome-wide association studies (GWAS). We first analyzed the relationship between allergic diseases and influenza from a holistic perspective, and then conducted multiple analyses on 3 specific allergic diseases and varying degrees of influenza. Multiple SNPs were utilized as IVs to represent genetic variations. Fig. 1 illustrates the workflow of the MR analysis. Our study design adheres to the core tenets of MR and employs MR techniques to improve the validity of epidemiological observational investigations (STROBE-MR).^21^

We obtained relevant data on allergic diseases, including AD, AR, and asthma, from the IEU OpenGWAS project. The GWAS of allergic diseases involved 360,838 individuals, comprising 180,129 cases of asthma, hay fever, or eczema, and 180,709 healthy controls. Concurrently, the sample size for AD comprised 481,299 participants, which included 6224 AD cases and 475,075 healthy controls. The sample size for asthma was 484,598, with 56,087 asthma cases and 428,511 healthy controls. Additionally, the sample size for hay fever or AR was 112,583, consisting of 25,486 cases and 87,097 healthy controls.

We collected GWAS summary statistics from the FinnGen dataset for 3 influenza datasets: All Influenza (4262 cases and 188,868 healthy controls), All influenza (not pneumonia) (2676 cases and 188,868 healthy controls), Influenza and Pneumonia (29,924 cases and 188,868 healthy controls). Here, we have provided specific definitions for these 3 different types of influenza datasets: ① All Influenza-All Influenza: Includes all cases of influenza, with and without pneumonia. ② All influenza (not pneumonia)-Regular Influenza: Cases of influenza without pneumonia (a subset of all influenza). ③ Influenza and Pneumonia-Severe Influenza: Cases of influenza with pneumonia, representing more serious instances (another subset of all influenza). The FinnGen study is a large-scale genomics initiative that has analyzed over 500,000 Finnish biobank samples. The detailed information regarding all involved traits is summarized in Table 1.

**Table 1:** Data sources and traits for MR analysis

Trait	Ancestry	Cases	Controls	Samplesize	No.SNPs	Dataset ID	Year
All influenza	Europeans	4262	188,868	193,130	16,380,378	finn-b-J10_INFLUENZA	2021
All influenza (not pneumonia)	Europeans	2676	188,868	191,544	16,380,370	finn-b-INFLUENZA	2021
Influenza and pneumonia	Europeans	29,924	188,868	218,792	16,380,466	finn-b-J10_INFLUPNEU	2021
Allergic disease	Europeans	180,129	180,709	360,838	8,133,670	ebi-a-GCST005038	2017
Atopic dermatitis	Europeans	6224	475,075	481,299	24,185,642	ebi-a-GCST90018784	2021
Asthma	Europeans	56,087	428,511	484,598	9,587,836	ebi-a-GCST90038616	2021
Hay fever or allergic rhinitis	Europeans	25,486	87,097	112,583	9,851,867	ukb-b-7178	2018

### Instrument selection

To ensure that the screened SNPs are strongly correlated with exposure factors, we established the following screening criteria. First, we set a significance threshold of p < 5.0 × 10ˆ–8 to identify significantly correlated SNPs. Next, we applied a linkage disequilibrium (LD) threshold of R^2^ < 0.001 and an aggregation distance of 10,000 kb to enhance randomness. To further ensure strong correlation, we required an F-statistic greater than 10 to exclude weak IVs. The F-statistic was calculated using the beta coefficient, standard error (SE), and p-value (p) for each SNP, following formula F = Beta^2^/SE^2^.^22^ Additionally, we computed R^2^ using the formula R^2^ = 2 × EAF × (1 - EAF) × Beta^2^, which represents the proportion of variability in the exposure factor explained by each IV.^23^ In this equation, EAF, Beta, and SE denote the allele frequency, effect size, and standard error of each SNP, respectively, all of which are associated with the exposure factor.

When allergic disease (asthma, hay fever or eczema) was used as exposure, we obtained 74 related independent SNPs (Table S1); When AD, asthma, and AR are exposure factors, we use the same criteria. Finally, we identified 24 independent SNPs associated with AD, 122 independent SNPs associated with asthma, and 33 independent SNPs associated with AR from the GWAS dataset (Table S1). In the harmonization process, palindromic SNPs with ambiguous strand identification and ambiguous SNPs with non-concordant alleles were excluded.

### Statistical analyses

To minimize the effects of horizontal pleiotropy, we first corrected outliers by employing the MR pleiotropy residual sum and outlier (MR-PRESSO) method with the number of distributions set at 1000. Then, horizontal pleiotropy was detected by the Egger intercept test, and P < 0.05 was considered significant. The I^2^ statistics and p value of Cochran's Q-statistics were applied to test for heterogeneity. In general, if p > 0.05 or I^2^ < 0.25, it is considered that no significant heterogeneity existed.^24^ Four complementary methods of MR analysis were performed. In the absence of heterogeneity and pleiotropy, a random-effects model of IVW-RE estimates was preferred. IVW-RE was also chosen when there was only heterogeneity and no pleiotropy.^25^ The consistency of the results obtained by the 4 methods can reduce the occurrence of false positive events. The results of IVW-RE will be inspected by leave-one-out sensitivity analysis to increase reliability. The relationships between allergic diseases and influenza were quantified as odds ratios (OR) and their corresponding 95% confidence intervals (CI). All MR analyses were conducted in R software (version 4.3.0). Power calculations for MR estimates were conducted using the binary outcomes tool available on the website (http://shiny.cnsgenomics.com/mRnd/). In addition, we will screen the selected SNPs (195 SNPs after removing duplicates) in dbSNP (NCBI): https://www.ncbi.nlm.nih.gov/snp/、GeneCards: https://www.genecards.org/、OMIM: https://www.omim.org/. Search for and further analyze the location, gene name, molecular function, and related phenotypes of SNPs based on website information.

## Results

### Evaluation of IVs

Table S1 displays the evaluation results of the IVs incorporated in our research. The F-statistics for all SNPs exceeded 10, consequently minimizing the potential for weak IV bias. The statistical power ranged from 0.03 to 1 in the analyses, with comprehensive summary details provided in Table S3. Unfortunately, certain outlier SNPs were identified and subsequently eliminated from the multiple causal analyses through the employment of the MR-PRESSO method, as detailed in Table S2. A thorough description of the outlier SNPs can be found in Tables S5 and S6. The characteristics of each SNP employed for the Mendelian randomization analysis are concisely delineated in Table S7. Table S8 lists detailed information on the SNP locations, gene names, and molecular functions involved in MR analysis, while Table 2 displays part of important SNP information for this study.

**Table 2:** Molecular functional information of SNPs related to allergic diseases, AR, and asthma

Number	SNP	Gene: Consequence	Molecular Function (part)
1	rs1059513	STAT6: Non coding transcript variant NAB2 : 500B downstream variant	STAT6: Signal transducer and activator of transcription 6, specifically activated by IL4 and IL13, overlapping D12S1644, with several alternatively spliced variants, expressed at higher levels in pancreatic beta cells NAB2: NGFI-A (nerve growth factor induced clone A) binding protein 2 (ERG2 binding protein) highly expressed in brain and thymus, with an alternatively splicing isoform missing exon 3, and in vascular smooth muscle cell in response to injury
2	rs12625547	NFATC2: Intron variant	Nuclear factor activated T cells c2, pre-existing, component, related to the NFKB/REL proteins and forming cooperative complexes with FOS and JUN on DNA, calcineurin dependent, involved in expression of genes collectively coordinating immune response
3	rs3024665 rs3024664	IL4R: Intron variant	Interleukin 4, receptor (see IL2RG), expressed on T-cell, with a polymorphism associated with increased signaling and susceptibility to atopy asthma and hyper IgE syndrome
4	rs56375023 rs72743461 rs56062135	SMAD3: Intron variant	Drosophila mad (mothers against dpp) related gene JV15-1, critical mediator of the TGFB signaling pathway, sequence specific transcriptional activator, physically interacting with AP1 family members: JUNB, JUNC and JUND and interacting with response elements to mediate transcriptional activation of target genes, involved in TGFB dependent regulation of steroidogenesis
5	rs5743618	TLR1: Missense variant	Drosophila transmembrane receptor toll homolog 1, ubiquitously expressed, with at least 2 alternatively spliced isoforms, tissue specific, maybe involved in morphogenesis and immune defense
6	rs6881706	IL7R: Non coding transcript variant	Interleukin 7, receptor (see IL2RG), with a membrane bound and an alternatively spliced secreted form
7	rs80064395	NRROS: Intron variant	Key regulator of transforming growth factor beta-1 (TGFB1) specifically required for microglia function in the nervous system, required for activation of latent TGF-beta-1 in macrophages and microglia, TGF-beta-1 activation mediated by LRRC33/NRROS is highly localized, indirectly plays a role in toll-like receptor (TLR) signaling
8	rs848 rs20541	rs848: IL13 : 3 prime UTR variant rs20541: IL13: Missense variant	Interleukin 13, expressed in activated T cell, important switch factor directing Ig-E synthesis
9	rs2227472	IL22 : 2 KB upstream variant	Cytokine that plays a critical role in modulating tissue responses during inflammation, plays an essential role in the regeneration of epithelial cells to maintain barrier function after injury and for the prevention of further tissue damage
10	rs10912564	TNFSF4: Intron variant	Tumor necrosis factor (ligand) superfamily, member 4, tax-transcriptionally activated glycoprotein1, human OX-40 ligand, expressed in T cell infected by HTLV1
11	rs2070901	FCER1G: 2 KB upstream variant	Immunoglobulin E, high affinity fc receptor, gamma polypeptide, expressed in most cells, homodimerizing
12	rs34173062	SHARPIN: Missense variant MAF1 : 2 KB upstream variant	Sharpin: Component of the LUBAC complex which conjugates linear polyubiquitin chains in a head-to-tail manner to substrates and plays a key role in NF-kappa-B activation and regulation of inflammation MAF1: Plays a role in the repression of RNA polymerase III-mediated transcription in response to changing nutritional, environmental and cellular stress conditions to balance the production of highly abundant tRNAs, 5S rRNA, and other small non-coding RNAs with cell growth and maintenance. Also plays a key role in cell fate determination by promoting mesorderm induction and adipocyte differentiation
13	rs41283642	TGFBR1 : 3 prime UTR variant	Transforming growth factor beta, receptor type I, regulating cell cycle progression, including 2 variants TGFBR1, 6A and TGFBR1, 10A, inactivated in pancreatic and biliary cancers, deleted in cutaneous T cell lymphoma
14	rs4749894	IL2RA: Intron variant LOC124902368: Intron variant	IL2RA: Interleukin 2, low affinity receptor, alpha chain 45 kDa, expressed in T cell, B lymphoblast lines, peripheral blood monocyte LOC124902368: None
15	rs74466521	IL4: Intron variant	B-cell growth factor 1, 12 kDa, released from T cell after lectin or antigen stimulation. Interleukin 4, 15 kDa, B-cell stimulating factor 1, important switch factor directing Ig-E synthesis
16	rs1898671	TSLP: Intron variant	Cytokine that induces the release of T-cell-attracting chemokines from monocytes and, in particular, enhances the maturation of CD11c (+) dendritic cells. Can induce allergic inflammation by directly activating mast cells. May act as an antimicrobial peptide in the oral cavity and on the skin.
17	rs2066362	IL33: Intron variant	Cytokine that binds to and signals through the IL1RL1/ST2 receptor which in turn activates NF-kappa-B and MAPK signaling pathways in target cells. Involved in the maturation of Th2 cells inducing the secretion of T-helper type 2-associated cytokines

### Causal effect of allergic diseases on influenza

**Fig. 2:**
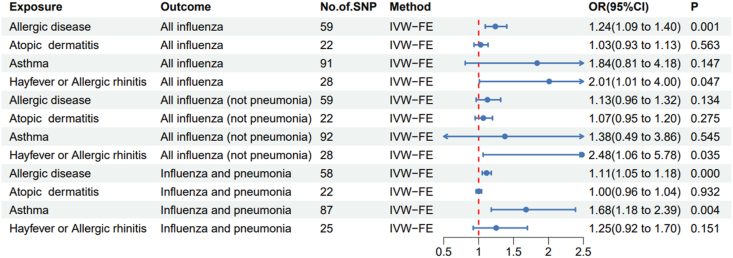
Causal estimation of disease and influenza

**Fig. 3:**
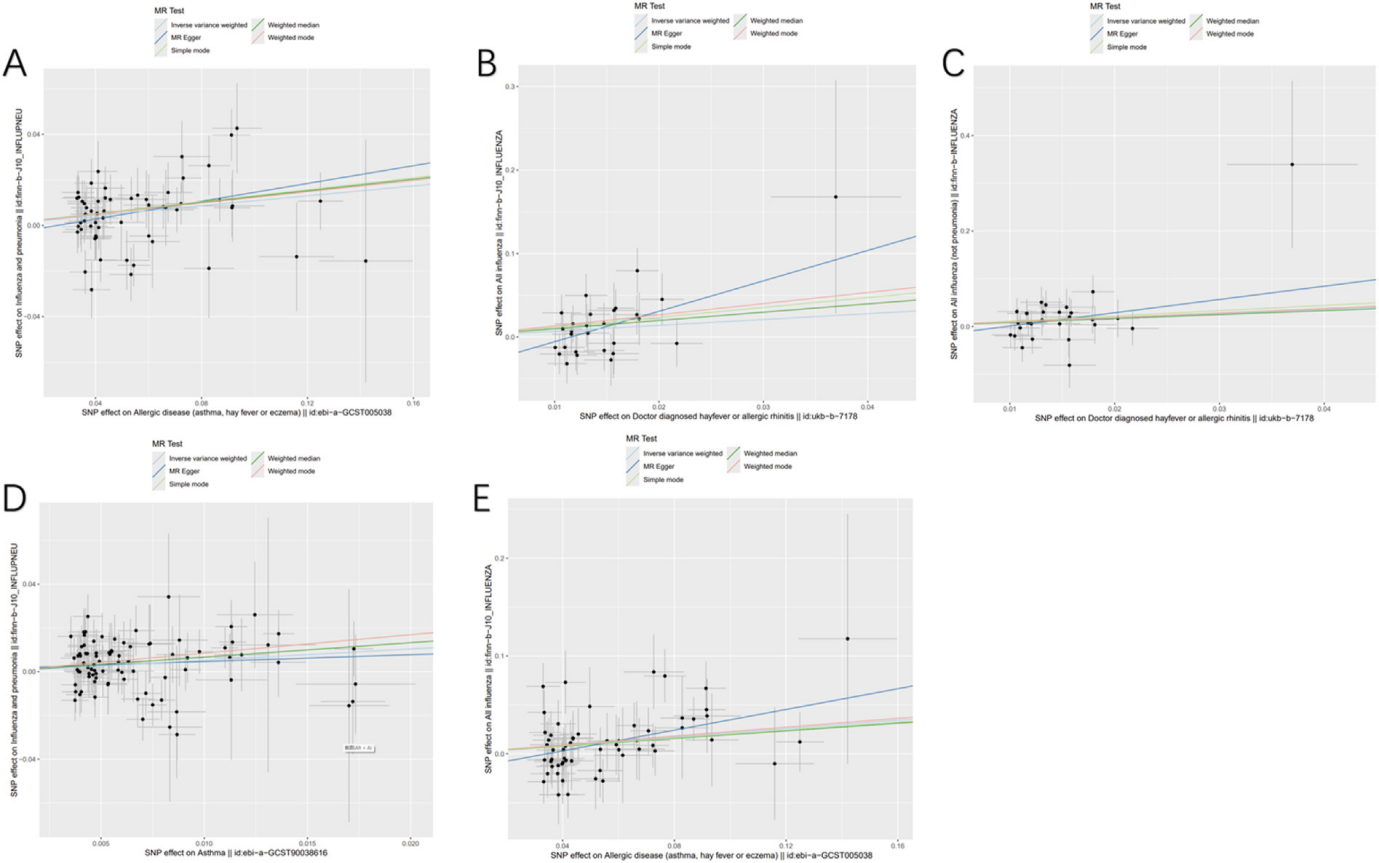
Scatter plots of MR analyses. (A) Allergic disease on influenza and pneumonia; (B) Hay fever or allergic rhinitis on all influenza; (C) Hay fever or allergic rhinitis on all influenza (not pneumonia); (D) Asthma on influenza and pneumonia; (E) Allergic disease on all influenza.

The MR analysis results of using IVW-RE method to evaluate the causal effects of allergic diseases on influenza are shown in Fig. 2. The random effects model can avoid the influence of heterogeneity. In a total of 12 causal analyses, we did not find significant horizontal pleiotropy through Egger intercept test (Table S2). According to the results of IVW-RE, we found significant correlations between allergic disease (asthma, hay fever, or eczema) and all influenza (OR = 1.24, 95% CI = 1.09–1.40, p = 0.001) and influenza (influenza and pneumonia) (OR = 1.11, 95% CI = 1.05–1.18, p = 2.26E-4); There is a correlation between AR (hay fever or allergic rhinitis) and both influenza (All influenza)(OR = 2.01, 95% CI = 1.01–4.00,p = 0.047)and regular influenza (All influenza (not pneumonia))(OR = 2.48, 95% CI = 1.06–5.78,p = 0.035); There is a correlation between asthma and severe influenza (influenza and pneumonia) (OR = 1.68, 95% CI = 1.18–2.39, p = 0.004). Allergic diseases, AR, and asthma are risk factors for influenza. The scatter plots of MR analysis for the above 5 groups of diseases are shown in Fig. 3. Table S4 summarizes the results of other MR methods. The sensitivity analysis of all scatter plots, funnel plots, and forest plots using the leave-one method can be found in the graphs S1–S4.

## Discussion

At present, to our knowledge, it is the first study to comprehensively evaluate the causal relationship between allergic diseases, AD, AR, and asthma as exposures, and influenza, regular influenza and severe influenza as outcomes, through the application of a dual sample MR analysis method. The analysis showed that allergic disease (asthma, hay fever or eczema) was significantly correlated with influenza (All influenza) and severe influenza (Influenza and pneumonia), respectively; There is a correlation between AR (hay fever or allergic rhinitis) and both influenza (All influenza) and regular influenza (All influenza (not pneumonia)); There is a significant correlation between asthma and severe influenza (Influenza and pneumonia). Our findings are supported by rigorous calibration and multiple sensitivity analysis. In particular, statistical tests have shown that in most positive results, values exceeding 60% indicate robust statistical power at a P-value threshold of 0.05.

The genes associated with SNPs related to allergic diseases, as listed in Table 2—such as STAT6, interleukin-13 (IL-13), interleukin-4 (IL-4), and FCER1G—hold significant relevance for understanding the genetic background, pathogenesis, treatment strategies, and molecular mechanisms of allergic diseases and influenza. These findings provide important references for the present study. Furthermore, SNPs that have not yet been clinically reported are expected to uncover new biological molecular mechanisms in future research. The early response to allergies is triggered by the localization and signal transduction of high-affinity Fc ε receptors on mast cells, as well as other cells, that bind to antigen-loaded IgE.^17^^,^^26^^,^^27^ In contrast, late-stage reactions, including airway hyperresponsiveness (AHR), involve the recruitment of various white blood cells. A common consensus among the subtypes of allergic reactions is that the initial triggering factor occurs in bronchial epithelial cells.^28^ The airway epithelium actively secretes cytokines that attract and activate immune cells, thereby providing a barrier and immune defense against foreign substances.^29^^,^^30^ Dendritic cells (DCs) dispersed within the epithelial layer capture inhaled allergens and are activated by these cytokines, initiating adaptive immune responses.^31^^,^^32^

Asthma and influenza are distinct diseases from an immunological perspective; however, the immune responses triggered in the pathogenesis of both conditions are complex and interconnected. Viral replication in the respiratory system can damage epithelial cells, leading to cell death, as these cells represent the first line of defense against invading pathogens.^10^ The primary immune response is initiated by epithelial cells and resident immune cells, resulting in the activation of the adaptive immune response, which more effectively inhibits viral replication.^33^ During IAV infection, cytokines related to wound repair [transforming growth factor-beta (TGF-β)], homeostasis interleukin-10 (IL-10), and allergy (IL-13) are also released.^34^, ^35^, ^36^ These cytokines enhance anti-influenza immune responses; however, their sustained presence in the lungs may prolong viral pneumonia^37^ and increase susceptibility to bacterial infections and asthma.^38^ Local interferon (IFN) signaling plays a critical role in suppressing viral replication. Paradoxically, even in the absence of IFN signaling, antiviral immune pathways may occur.^39^ Activated DCs present viral antigens to naive/memory T cells, initiating an adaptive immune cascade that includes activated B cells producing antibodies. Cytotoxic T cells and natural killer cells are pivotal in controlling infections and facilitating viral clearance.^40^^,^^41^

T-helper type 1/T-helper type 2 (Th1/Th2) immune imbalance is a crucial factor contributing to airway allergic disease.^42^ Th1 primarily orchestrates cellular immunity by secreting cytokines like interferon-γ (IFN-γ) and interleukin-2 (IL-2). IFN-γ plays a pivotal role in suppressing the differentiation of Th2 cells and inhibiting IgE synthesis by B cells, thereby reducing allergic reactions.^43^ On the other hand, Th2 predominantly governs humoral immunity by releasing cytokines such as IL-4/interleukin-5 (IL-5).^44^ IL-4, in particular, promotes Th2 cell differentiation, resulting in the secretion of Th2-associated cytokines (IL-4/IL-5/IL-13). Additionally, IL-4 inhibits Th1 cell proliferation, amplifies mast cell degranulation, and exacerbates allergic responses. Bronchoalveolar lavage fluid from patients with allergic asthma (AAS) exhibits elevated levels of Th2 cytokines,^30^ particularly IL-5, which is strongly linked to eosinophilic inflammation.^3^ Eosinophils serve as a significant source of various cytokines, including IL-13.^45^ IL-13 contributes to the pathophysiology of the disease by enhancing AHR and promoting excessive mucus production.^46^^,^^47^ Upon allergen exposure, antigen-presenting cells secrete IL-10, which activates downstream CD4^+^ T cells to differentiate into Th2 cells.^48^ Perturbations in the equilibrium between Th1 and Th2 populations significantly increase the susceptibility to allergic diseases.^49^ Notably, research has indicated that influenza virus can impede the recruitment of eosinophils and Th2 cells to the airways following allergen exposure.^50^ The reduction in characteristic Th2 immunity following influenza virus infection is paralleled with heightened levels of IFN-γ and CD8^+^ T cells.^51^, ^52^, ^53^ After allergic patients are attacked by influenza, their bodies may prioritize fighting against the influenza virus rather than triggering allergic reactions, shifting from Th2 dominant to Th1 dominant responses, which may reduce allergic reactions during infection.

The impact of previous asthma on the severity of influenza virus infections remains a topic of debate. Our research findings indicate that allergic diseases, including asthma, are significant risk factors for influenza infection. During the pandemic, patients who have not received long-term treatment to manage their asthma symptoms exhibited a higher incidence of pH1N1 infections.^54^ Asthma was recognized as a condition that complicates the pathogenesis of influenza, contributing to increased hospitalization rates for pH1N1, with approximately 25% of hospitalized patients requiring admission to intensive care units (ICUs).^55^ Interestingly, despite the association between asthma and a higher hospitalization rate during influenza, asthmatic patients who are hospitalized tend to experience less severe illness and lower mortality rates than those without asthma.^56^ Some studies suggest that the reasons for the above results may be related to the use of steroids, but corticosteroid treatment also increases the risk of pH1N1 infection and death.^57^, ^58^, ^59^ In addition, many hospitalized patients have multiple underlying diseases, and IAV infection can further worsen pre-existing diseases, indicating that the number of severe cases caused by influenza may be underestimated.^60^ The inconsistency in the number of severe cases mentioned above still requires further in-depth research.

The severe consequences of influenza infection are often associated with secondary bacterial infections.^61^ Notably, many individuals who died during influenza pandemics exhibited coexisting bacterial pneumonia, with a significant proportion of deaths during the 1918 influenza pandemic attributed to secondary complications from pneumococcal infection. Compared to pH1N1 patients without pneumonia, those who developed acute pneumonia due to pH1N1 exhibited significantly elevated levels of Th2 cytokines (IL-4, IL-5, and IL-13) in their serum.^62^^,^^63^ Asthma patients usually have a baseline state of Th2 inflammation, such as IL-4/IL-13 driving IgE production, IL-5 mediating eosinophil activation, and influenza infection may further amplify Th2 response, forming a “double blow”.^42^ This may lead to the already imbalanced Th1/Th2 balance in asthma patients being more likely to develop into a “Th2 storm” during infection, resulting in a doubled risk of pneumonia.^44^ Interestingly, primary bronchial epithelial cells derived from asthmatic donors demonstrated resistance to pH1N1-induced cell pathology, whereas primary bronchial epithelial cells from healthy donors did not.^64^ In a chronic AAS mouse model sensitized with ovalbumin (OVA), the population of T cells producing IFN-γ in the lungs was significantly reduced. Mice with asthma demonstrated increased susceptibility to influenza virus infection compared to control mice, exhibiting lower survival rates and higher viral titers in the lungs.^65^ Additionally, research has indicated that neutropenic asthma is associated with impaired type I IFN production, and the emergence of influenza virus variants correlates with the severity of the disease in neutropenic asthma models.^66^ These findings suggest that a subset of asthma patients may be more vulnerable to severe influenza and may serve as a potential source for new influenza virus variants. Consequently, in a mouse model of influenza virus infection following airway allergic disease, the effects of airway allergic disease could be either beneficial or detrimental.^67^ The interaction between the influenza virus and allergic hosts is influenced by various factors, including asthma subtype, host immune status, underlying comorbidities, and airway conditions.^17^^,^^68^ Healthy airways, allergen-sensitized airways, and airways recently exposed to allergens represent 3 distinct states, each of which may respond differently to IAV infection.^69^

The research results indicate a causal relationship between AR and influenza. The various manifestations of AR are strongly influenced by genetic factors, with multiple genes and associated transcription factors implicated in the pathogenesis of this condition.^70^ These include candidate genes related to IgE, significant transcription factors, cytokines, and T cell surface antigens.^19^ Active and effective treatment of AR can prevent and alleviate asthma attacks. Studies have shown that toll-like receptor expression is impaired, and rhinovirus replication is increased in the nasal epithelial cells of patients with AR.^71^ Compared to individuals without AR, those with nasal symptoms exhibit more severe influenza-related symptoms, heightened susceptibility of the allergic nasal mucosa to IAV, and increased viral load, which may be associated with damage induced by type III IFN.^72^

At present, there is no effective curative treatment for AD, AR, or AAS. Corticosteroids and antihistamines are commonly used to treat these 3 allergic diseases; but symptoms often rebound after stopping the medication.^32^ Respiratory viruses interact with allergens and other microorganisms, promoting the development of recurrent virus induced wheezing and asthma through various mechanisms such as increased recruitment of inflammatory cells and promotion of cytokine production. The acute phase of influenza not only induces respiratory inflammation and tissue damage but also exacerbates unrelated local allergic reactions through a Th2 response. Influenza vaccination may not directly prevent or reduce the severity of influenza infections; however, its immune regulatory effects are beneficial.^73^ The World Health Organization (WHO) recommends that all individuals receive an annual influenza vaccine, particularly vulnerable populations such as pregnant women, children, the elderly, and those with underlying health conditions.^74^, ^75^, ^76^ However, compliance with this recommendation remains low. While no causal relationship has been established between AD and influenza, a national cohort study indicated that influenza vaccination is associated with a reduced risk of asthma among patients with AD. Specifically, this study found that the adjusted hazard ratio for asthma between vaccinated and unvaccinated groups was 0.69 (95% CI = 0.55–0.87), and the cumulative incidence rate of asthma in vaccinated patients was notably lower.^71^ As the risk of asthma in vaccinated participants of all age and gender groups tends to decrease, the necessity of annual vaccination against asthma exacerbations is evident.^77^^,^^78^

Our research has 3 prominent characteristics. Firstly, in an observational environment, use GWAS from a large sample to simulate randomized controlled trials using MR methods. Secondly, our MR analysis includes both global analysis and subgroup analysis, which makes our research results more comprehensive and more reliable. Thirdly, various sensitivity analyses provide sufficient evidence for the stability and reliability of our research results.

However, our research still has some limitations. At first, the study only discovered unidirectional effects. Due to the absence of SNPs that are significantly correlated with the outcome, reverse detection is not feasible. It is advisable to pursue bidirectional estimation to further elucidate these relationships. Moreover, the data under analysis is derived from open databases and is aggregated. The participants in our study were all from European populations. Therefore, the findings cannot apply to other populations. In fact, influenza and allergic populations involve people from all over the world.

## Conclusion

This study revealed the potential causal relationship between allergic diseases and susceptibility to influenza through MR analysis. The main findings indicate that allergic diseases such as asthma and AR may significantly increase the risk of influenza infection through immune regulatory mechanisms such as Th2 type immune response activation and epithelial barrier dysfunction. The research findings have significant clinical and public health implications. Firstly, it is recommended to include patients with allergic diseases in the management of high-risk populations for influenza, prioritize seasonal vaccination, and strengthen early symptom monitoring. In addition, the research results provide mechanistic evidence to explain the phenomenon of increased hospitalization rates among allergy prone populations during influenza epidemics. This research may contribute to a deeper understanding of the pathogenesis of allergic diseases and influenza, and propose new treatment methods.

## Abbreviations

MR, Mendelian randomization; GWAS, genome-wide association studies; AD, atopic dermatitis; AR, allergic rhinitis; IVW-RE, inverse variance weighted random effects model; AAS, allergic asthma; IgE, immunoglobulin E; AHR, airway hyperresponsiveness; CLRDs, chronic lower respiratory diseases; HA, hemagglutinin; NA, neuraminidase; RSV, respiratory syncytial virus; RV, rhinovirus; IAV, influenza A virus; URI, upper respiratory tract infections; SNPs, single nucleotide polymorphisms; IVs, instrumental variables; LD, linkage disequilibrium; SE, standard error; p, p-value; MR-PRESSO, MR pleiotropy residual sum and outlier; OR, odds ratios; CI, confidence intervals; Th1, T-helper type 1; Th2, T-helper type 2; IL-2, interleukin-2; IL-4, interleukin-4; IL-5, interleukin-5; IL-10, interleukin-10; IL-13, interleukin-13; ICUs, intensive care units; DCs, dendritic cells; TGF-β, transforming growth factor-beta; IFN, interferon; IFN-γ, interferon-γ; OVA, ovalbumin.

## Data availability statement

The data for this study were sourced from publicly available databases. All datasets in the article are sourced from the following website: IEU OpenGWAS project, https://gwas.mrcieu.ac.uk/.

## Author contributions

HL and YPZ: Analyzed data and drafted the initial manuscript. LH and YJS: Reviewed references. JCG and WBS: Conceptualized, reviewed, and edited the manuscript.

## Statement on ethics

Given that all data were obtained from publicly accessible summaries of GWAS results and the studies involved had obtained approval from their institutional ethics review panels, no further ethical clearance was necessary.

## Consent for publication

This manuscript has not been published or presented elsewhere in part or in entirety, and is not under consideration by another journal. All the authors have approved the manuscript and agree with submission to your esteemed journal.

## Funding

This research was supported by the project “Unveiling the List of Commanders” from the Institute of Xin'an Medicine and the Modernization of Traditional Chinese Medicine at the Research Institute of Big Health, Hefei Comprehensive National Science Center (2023CXMMTCM009).

## Declaration of competing interest

The authors affirm that the study was carried out without any commercial or financial ties that may create a possible conflict of interest.

## Footnotes

Appendix A Supplementary data to this article can be found online at https://doi.org/10.1016/j.waojou.2025.101077.
